# Estimating genomic heritabilities at the level of family-pool samples of perennial ryegrass using genotyping-by-sequencing

**DOI:** 10.1007/s00122-015-2607-9

**Published:** 2015-09-25

**Authors:** Bilal Hassan Ashraf, Stephen Byrne, Dario Fé, Adrian Czaban, Torben Asp, Morten G. Pedersen, Ingo Lenk, Niels Roulund, Thomas Didion, Christian S. Jensen, Just Jensen, Luc L. Janss

**Affiliations:** Dept. of Molecular Biology and Genetics, Aarhus University, Blichers Allé 20, Postbox 50, 8830 Tjele, Denmark; Dept. of Molecular Biology and Genetics, Aarhus University, Forsøgsvej 1, 4200 Slagelse, Denmark; DLF-Trifolium A/S, Research Division, Højerupvej 31, 4600 Store Heddinge, Denmark

## Abstract

*****Keymessage***:**

**By using the genotyping-by-sequencing method, it is feasible to characterize genomic relationships directly at the level of family pools and to estimate genomic heritabilities from phenotypes scored on family-pools in outbreeding species.**

**Abstract:**

Genotyping-by-sequencing (GBS) has recently become a promising approach for characterizing plant genetic diversity on a genome-wide scale. We use GBS to extend the concept of heritability beyond individuals by genotyping family-pool samples by GBS and computing genomic relationship matrices (GRMs) and genomic heritabilities directly at the level of family-pools from pool-frequencies obtained by sequencing. The concept is of interest for species where breeding and phenotyping is not done at the individual level but operates uniquely at the level of (multi-parent) families. As an example we demonstrate the approach using a set of 990 two-parent F2 families of perennial ryegrass (*Lolium Perenne*). The families were phenotyped as a family-unit in field plots for heading date and crown rust resistance. A total of 728 K single nucleotide polymorphism (SNP) variants were available and were divided in groups of different sequencing depths. GRMs based on GBS data showed diagonal values biased upwards at low sequencing depth, while off-diagonals were little affected by the sequencing depth. Using variants with high sequencing depth, genomic heritability for crown rust resistance was 0.33, and for heading date 0.22, and these genomic heritabilities were biased downwards when using variants with lower sequencing depth. Broad sense heritabilities were 0.61 and 0.66, respectively. Underestimation of genomic heritability at lower sequencing depth was confirmed with simulated data. We conclude that it is feasible to use GBS to describe relationships between family-pools and to estimate genomic heritability directly at the level of F2 family-pool samples, but estimates are biased at low sequencing depth.

**Electronic supplementary material:**

The online version of this article (doi:10.1007/s00122-015-2607-9) contains supplementary material, which is available to authorized users.

## Introduction

Use of genomic data offers new possibilities to estimate genomic heritabilities by using relationships computed based on genomic information. In a human height study, Yang et al. (2010) estimated such a genomic heritability based on single nucleotide polymorphisms (SNPs) without the need for pedigree information. This can also be interesting for breeding material that is not handled as individuals, but in so-called “population-based” breeding systems that operate at the level of (multi-parent) family-pools. In these systems, family-pools are the basic unit for phenotyping, for development into commercial varieties, and for development of new “progeny” family-pools. Classical genetic concepts like pedigrees, genetic relationships and heritability cannot be used because they have individuals as the focal unit. Use of genomic information, however, would allow computing relationships directly at the family-pool level and to develop a genomic-based concept of heritability at the family-pool level, provided that a suitable genotyping for family-pool samples would be available.

An example of a species using “population based” breeding using family-pools is perennial ryegrass (*Lolium perenne L*.). *Lolium Perenne L.* belongs to the group of inter-fertile and largely outbred grass species (Wilkins 1991). Individual plants are of limited interest for breeding: as an outbreeder, individuals cannot be propagated into commercial varieties, and relevant phenotypes are scored in field plots were individuals cannot be distinguished (Connolly 2001). *Lolium Perenne* is the principal forage grass of N. Europe and is also grown widely in temperate climate zones worldwide for feeding livestock and/or amenity grass in sport grounds and residential areas. Generally, forage grasses have a relatively short history of formal breeding, which implies great variation within and among populations, and significant potential for genetic improvement (Conaghan and Casler 2011).

DNA sequence yields an unrivalled source of genetic information. Recent developments in next-generation sequencing (NGS) technologies have resulted in a continuous improvement in the throughput, speed, and cost to obtain genome sequences. These advancements have improved the potential to detect genetic variation that can be traced by a set of dense markers covering the whole genome. A robust genotyping approach known as genotyping-by-sequencing (GBS) developed by Elshire et al. (2011) reduces genome complexity by using restriction enzymes. GBS approaches using methylation sensitive restriction enzymes such as *ApeKI* can be used to target the low copy fraction of the genome (Elshire et al. 2011). These approaches can also be used in breeding populations of outbred heterogeneous varieties by estimating genome-wide allele frequency profiles in pooled samples (Byrne et al. 2013). Until recently, use of GBS has only been reported for studies where sequences were obtained on individuals (Peterson et al. 2014). To genotype family pools, GBS provides a direct way to measure allele frequencies at bi-allelic SNPs. Recently, it has also been shown that GBS data can be used directly without calling genotypes, which allows to use this technology for measurements of F2 family pools (Ashraf et al. 2014). Analysis of genomic variation is an essential part of plant genetic and crop improvement. Despite large genetic variability in perennial ryegrass, only a few investigations have reported variance components for traits measured in ryegrass (Conaghan and Casler 2011; Yamada et al. 2004), and are often based on phenotypes measured on individuals instead of pools. To date, no studies have reported genomic heritabilities in breeding populations of perennial ryegrass using GBS data.

Use of GBS also poses statistical challenges, for instance, genotyping error at low sequencing depth and missing values (Merrill 2013; Poland and Rife 2012). Sequencing depth is an important experimental variable in GBS because it determines the accuracy of genotype estimates or pool allele frequency estimates. In principle, increasing the sequencing depth will improve accuracy of the genotypes (Sims et al. 2014), but if total sequencing budget is fixed this implies reducing the number of samples which in turn reduces the power in a Genome Wide Association Study (GWAS) (Ashraf et al. 2014). Several studies have been conducted to investigate the effect of sequencing depth in GWAS (Garner 2011; Sims et al. 2014), but the effect of sequencing depth in relation to the estimation of genomic heritability has not yet been addressed. Ashraf et al. (2014) showed bias in allele-effect estimates when sequencing depth is low, and it is expected that bias also arises in genomic heritability estimates when using GBS data with low sequencing depth.

Many studies have investigated the importance of markers density in genomic selection (GS) of animal and plants (Habier et al. 2009; Heffner et al. 2009). In human height, Yang et al. (2010) found that a large proportion of genomic heritability can be traced by using a relatively small number of SNP markers. Similarly in a dairy cattle study, Jensen et al. (2012) showed that relatively large proportion of additive genetic variance may be traced by a limited number of markers in cases with a strong family structure. Use of SNPs from GBS data is under investigation in many crop species (Lin et al. 2014), but mainly in the context of genomic prediction. Effect of marker - density on the estimates of genomic heritability when using GBS data has not been investigated in previous studies on perennial ryegrass or other species.

The main objective of this study was to demonstrate feasibility of computing relationships directly at the level of family-pools from genotyping-by-sequencing. This allows taking family-pools as a unit of measurement with a genotype and a phenotype, and to compute heritabilities at the level of family-pool phenotypes as they arise in breeding populations of perennial ryegrass. To validate this approach, a large data set from a single year and single location is used and procedures are demonstrated for crown rust resistance and heading date as example traits. We also determine the effects of different sequencing depths and marker density on the estimates of genomic heritability and the results from experimental data were verified using stochastic simulation.

## Materials and methods

### Phenotypic data

The origin of the breeding material used in the current study has been described in detail by Fè et al. (2015), but for the current study families were re-phenotyped in a single year and single location at DLF Trifolium A/S Denmark. In a field trial 995 plots of F2 families were established during the autumn 2010, with two replicates per family.

Phenotypic data was scored at the plot level for each F2 family. Crown rust resistance was scored once during the growth season and measured by visual scoring of the field-plot on a 0 (maximum infection) to 9 (no infection) scale during the period of maximum infection. Heading date was also scored on the field plot and was defined as the day on which plants start showing on average at least one spikelet per tiller. It was expressed in days after 1st of May.

### Genomic data

Sequence data was obtained by genotyping-by-sequencing (GBS) of bulk samples of the families. Sampling and library preparation were carried out according to Byrne et al. (2013), and Elshire et al. (2011) and was based on a bulk sample of leafs from 200 to 500 plants per family. We prepared 16 libraries, each with up to 64 F2 families and sequenced each library on seven lanes of on an Illumina HiSeq 2000 (single-end). Top-up libraries were prepared to bulk up output for F2 families competing poorly within a library. A total of 995 F2 families were genotyped using GBS and the average number of reads obtained per F2 population after basic data filtering (see supplemental note 1) was 20.5 million. Data for each F2 family was aligned against a draft sequence assembly and 1,020,065 SNPs were identified (see supplemental note 1) with sequencing depth at a SNP ranging from 0 to 250 (upper limit) reads per family. Figure S1 (supplementary material) shows the average frequency and average sequencing depth of SNP markers, showing that SNPs with average depth more than 60 have a different range in allele frequencies, and allele frequencies often close to 1. Few SNP positions had greater than 60 reads and we suspect that these reads may be originating from plastid genomes or highly repetitive regions not captured in the draft assembly. We therefore decided to discard all SNPs with average depth more than 60. Further, SNPs with allele frequencies less than 0.02 and greater than 0.98 were removed. After this filtering on depth and frequency, 728,359 SNPs were available for analysis. SNPs were split into five different subgroups based on average SNP sequencing depth (0–10, 10–20, 20–30, 30–40 and 40–60), with numbers of SNPs in each group as shown in Table 1. These groups of SNPs were the basis for constructing GRM based on SNPs with different sequencing depth. Five of the families showed very high genotype missing rate (>50 %) and were removed, leaving 990 families in the final data set.

**Table 1:** Number, mean diagonal, mean off-diagonal and their standard errors (in parentheses) for Genomic Relationship Matrices constructed from SNPs’ subgroups based on different sequencing depths

Sequencing depth	# SNPs	Diagonal	Off-diagonal
0–10	164,572	2.666 (0.013)	−0.0026 (0.000080)
10–20	337,011	2.409 (0.014)	−0.0024 (0.000091)
20–30	131,592	1.977 (0.013)	−0.0020 (0.000091)
30–40	60,147	1.640 (0.012)	−0.0016 (0.000084)
40–60	35,037	1.296 (0.011)	−0.0013 (0.000068)

### Genomic relationship matrices (GRM) using different sequencing depth

To investigate the effect of sequencing depth on genomic heritability estimates, we constructed GRMs based on SNPs with different sequencing depth (0–10, 10–20, 20–30, 30–40 and 40–60). There are several versions of GRMs in various studies, e.g., Legarra and Misztal (2008) and Forni et al. (2011), but here we follow the ‘method 1’ from VanRaden (2008) with modifications to accommodate for F2 family pools instead of single individuals. We also added the correction for missing rate from VanRaden (2008, p 4420). Consider a matrix comprised of allele frequency estimates from GBS data with SNPs in rows and samples in columns, *X*_*ij*_ being the allele frequency at SNP *i* in family *j*, and *M* is the matrix centered by SNP mean frequencies, $M = \left\{ {M_{ij} } \right\} = \left\{ {X_{ij} - \bar{X}_{i} } \right\}$. Missing SNP data is substituted by the mean SNP frequency. This amounts to a mean-imputation for missing genotypes. Then the G-matrix is:

$$G = \frac{{M^{\prime } M}}{K}$$

Here *K* is taken as 0.25 $\sum {\bar{X}_{i} \left( {1 - \bar{X}_{i} } \right)}$, which is the sum of expected SNP variances when using frequencies in families (Ashraf et al. 2014) and when inbreeding is absent. Because the F2 families derive from F1 × F1 full sib intercrosses, the F2 families are expected to be 25 % inbred and to have *G* diagonals of 1.25 after this scaling. The inverse of *G* was computed by first computing the eigen-decomposition $G = E_{v} \varLambda E_{v} '$. Typically a *G*-matrix is singular, with one last zero eigenvalue due to the centering procedure. The last (close to) zero eigenvalue from the *G*-matrix was increased to a value just below the one-by-last eigenvalue, and subsequently the inverse of *G* was computed as:

$$G_{\text{inv}} = E_{v} '\varLambda^{ - 1} E_{v}$$

These computations were performed in R version 3.0.2 (R development Core Team 2014).

### GRMs at lower and equal SNP densities within sequencing depth

Dividing the SNPs in groups with different sequencing depth yielded different group sizes. To compare results using constant number of SNPs in each group, and to study the effect of using lower numbers of SNPs within sequencing depth random sub-samples were made within each group of SNPs of 5, 10, 15, 20, 25, 30 and 35 K SNPs. The size 35 K corresponds to a number of SNPs just below the size of the smallest group. Again GRMs were constructed using these groups of SNPs.

### REML variance component estimation

Phenotypic data was analyzed by REstricted Maximum Likelihood (REML) using the average information (AI-REML) algorithm in the DMU multivariate mixed model package (Madsen and Jensen 2000). The raw phenotypic data was analyzed which includes two replicates (plots) per family. The data were analyzed using the model:

1 $$y \, = \, Xt \, + Zg \, + \, Zf \, + \, Wp \, + e$$

where *y* is a vector of phenotypic observations; *X* is a design matrix for trials, which are blocks of 36 plots in the field; *t* is a vector of trial effects; *Z* is a design matrix relating observations to families with two plots for most families; *g* is a vector of genomic breeding values for families with *g* ~ *N* (0, *Gσ*_*g*_^*2*^), with *σ*_*g*_^*2*^ being the genomic variance and *G* is the genomic relationship matrix; *f* is a vector of remaining uncorrelated family effects with *f* ~ *N* (0*, Iσ*_*f*_^*2*^), where *σ*_*f*_^*2*^ is the variance due to uncorrelated family effects, *W* is a design matrix relating families to the parent-populations from which their parents were sampled, *p* is a vector of random effects of parent-populations with *p* ~ *N* (0, *Iσ*_*p*_^*2*^) where *σ*_*p*_^*2*^ is the variance explained by parent populations, and *e* is vector of random residuals e ~ *N* (0*, Iσ*_*e*_^*2*^), with *σ*_*e*_^*2*^ corresponding to the variance of residuals. Note that both *g* and *f* are family effects, with *g* correlated between families according to estimated genomic relationships, and *f* uncorrelated between families. The uncorrelated family effect is included to capture any covariance between family repeats that is not captured by the genomic relationship matrix; this covariance could include non-additive genetic effects and specific family by environment interaction effects. Total explained variance in the above model was obtained as:

$$\text{var} \left( y \right) = G\sigma_{g}^{2} + I\sigma_{f}^{2} + WW^{'} \sigma_{p}^{2} + I\sigma_{e}^{2}$$

Total explained variance is evaluated from this expression by using the average diagonal value of the used GRM for *G*, and using the value of 2 for WW′ because in the data considered every family had two parents from two parent-populations.

From the variance components, two narrow-sense heritabilities and broad sense heritability were calculated as:

$$h_{G}^{2} = {\raise0.7ex\hbox{${\sigma_{g}^{2} }$} \!\mathord{\left/ {\vphantom {{\sigma_{g}^{2} } {\text{var} (y)}}}\right.\kern-0pt} \!\lower0.7ex\hbox{${\text{var} (y)}$}}$$

$$h_{G + PP}^{2} = {\raise0.7ex\hbox{${\left( {\sigma_{g}^{2} + 2\sigma_{p}^{2} } \right)}$} \!\mathord{\left/ {\vphantom {{\left( {\sigma_{g}^{2} + 2\sigma_{p}^{2} } \right)} {\text{var} (y)}}}\right.\kern-0pt} \!\lower0.7ex\hbox{${\text{var} (y)}$}}$$

$$H^{2} = {{\left( {G\sigma_{g}^{2} + 2\sigma_{p}^{2} + \sigma_{f}^{2} } \right)} \mathord{\left/ {\vphantom {{\left( {G\sigma_{g}^{2} + 2\sigma_{p}^{2} + \sigma_{f}^{2} } \right)} {\text{var} (y)}}} \right. \kern-0pt} {\text{var} (y)}}$$

where $h_{G}^{2}$ is a narrow sense heritability based on genomic relationships only, $h_{G + PP}^{2}$ is a narrow sense heritability based on genomic and parent-population variance, and *H*^2^ is broad sense heritability including all variance component related to families.

### Simulation studies

To verify the principle and possible biases of estimating genomic heritabilities using family-pool samples a simulation study was performed creating artificial data sets with 500 families and 1000 SNP markers. The SNP markers were considered independent, implying that ancestral Linkage Disequilibrium between the markers is ignored. For each SNP marker a population frequency was drawn from a uniform distribution between 0.05 and 0.95. The family-pools considered were F2 pools from two diploid parents. As derived in Ashraf et al. (2014) the “genotype” of such a pool can be considered as a tetraploid genotype, with frequencies within the pools in quarters, and this was used to directly generate the true pool-genotypes. Subsequently, the effect of using GBS for genotyping the pool sample was simulated, by considering that each pool-genotype was estimated by obtaining 5, 15 or 25 sequence-reads on each pool. The allele-counts in the sequence-reads then follow a binomial distribution of size 5, 15, or 25 with probability the true pool genotype; the GBS estimate of the pool genotype is then the proportion of successes in this binomial sampling. Phenotypes were generated by considering 5, 10, 50, 100, 500 and 1000 of the SNP markers (of 1000 total) to be a QTL, and QTL effects were drawn from a standard Normal distribution. The simulated total genetic values were standardized to have a variance of 1. Data sets with one replicate per family were generated according to the model:

$$y_{i} = \mu + \sum\limits_{k = 1}^{Nq} {q_{ki} a_{k} + e_{i} }$$

and data sets with two replicates per family were generated according to the model:

$$y_{ij} = \mu + f_{i} + \sum\limits_{k = 1}^{Nq} {q_{ki} a_{k} + e_{ij} }$$

where *y*_*i*_ is a single phenotype on family *i*, *y*_*ij*_ are repeated phenotypes for *j* = 1,2 on family *i*, *μ* is an overall mean, *Nq* is the number of QTL, *q*_*ki*_ is the genotype frequency at QTL *k* for family *i*, *a*_*k*_ is effect of QTL *k*, and *e*_*i*_ and *e*_*ij*_ are model residuals. For analysis of these simulated data sets GRM’s were made using all simulated markers following the same procedures as for the empirical data. For each presented scenario 50 data sets were generated and results were averaged over replicated data sets. All analyses followed the same REML procedures as for the empirical data analyses, except for leaving out the effect of parent-populations, and obtaining narrow sense genomic heritability $\left( {h_{G}^{2} } \right)$ for the scenario with one replicates per family, and narrow sense genomic and broad sense heritability (*H*^2^) for the scenario with two replicates per family.

## Results

### *G*-matrices at different sequencing depth

**Fig. 1:**
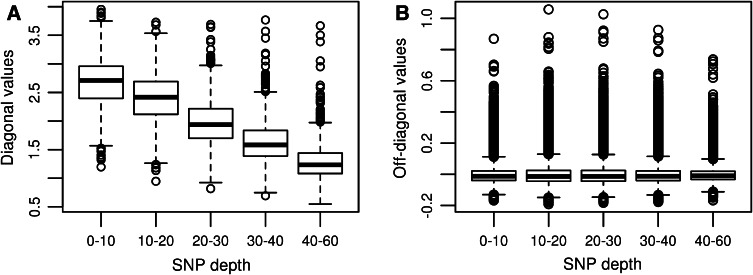
Box plots for diagonal values (*left panel*) and off-diagonal values (*right panel*) of genomic relationship matrices computed from SNPs with different sequencing depths

Table 1 shows the impact of sequencing depth on diagonal and off-diagonal elements of the GRMs. The matrices were scaled to have expected diagonals of 1.25 (reflecting 25 % inbreeding) for these F2 families, and with high sequencing depth diagonals of GRMs approach the expected value. However, at low sequencing depth, diagonal values are much higher than the expected value; at depth 0–10 the average diagonal value was 2.666. Off-diagonals, in contrast, were hardly affected by sequencing depth. Box plots of the diagonal and off-diagonal values in the genomic relationship matrices with different sequencing depth are shown in Fig. 1.

### Genomic heritabilities

Table 2 presents results of the REML analyses showing three heritabilities for two traits, using GRMs based on SNPs with different sequencing depth. The broad sense heritabilities are virtually constant for crown rust resistance and very similar for heading date across sequencing depth, showing that the total variance explained by families is not affected by sequencing depth. However, results show that sequencing depth has a significant impact on the amount of variance that is captured by the genomic component $\left( {h_{G}^{2} } \right)$; at low sequencing depth ${h_{G}^{2} }$ captures a smaller portion of the total family variance. The effect of parent-population only partly compensates for this, and total narrow sense heritability based on the genomic and parent-population components $h_{G + PP}^{2}$ is still lower at low sequencing depth.

**Table 2:** Estimated narrow sense heritability based on genomic relationships ($h_{G}^{2}$), narrow sense heritability based on genomic relationships and parent population ($h_{G + PP}^{2}$) and broad sense heritability (*H* ^2^) for crown rust resistance and heading date using pooled family data and SNP sets with different sequencing depths

Sequencing depth	Crown rust resistance			Heading date		
	$h_{G}^{2}$	$h_{G + PP}^{2}$	*H* ^*2*^	$h_{G}^{2}$	$h_{G + PP}^{2}$	*H* ^*2*^
0−10	0.17 (0.057)	0.22 (0.051)	0.60 (0.096)	0.07 (0.030)	0.51 (0.051)	0.69 (0.068)
10−20	0.19 (0.057)	0.23 (0.050)	0.60 (0.081)	0.09 (0.033)	0.51 (0.050)	0.68 (0.066)
20−30	0.25 (0.061)	0.27 (0.053)	0.61 (0.060)	0.11 (0.037)	0.51 (0.050)	0.68 (0.057)
30−40	0.23 (0.064)	0.26 (0.057)	0.60 (0.045)	0.17 (0.043)	0.52 (0.048)	0.66 (0.050)
40−60	0.33 (0.079)	0.35 (0.070)	0.61 (0.032)	0.22 (0.052)	0.58 (0.050)	0.66 (0.042)

### Genomic heritabilities with smaller and equal SNP sets

The results in Table 2 are based on GRM with different number of SNPs in each level of sequencing depth. To compare results at smaller and equal SNP density, heritabilities were computed using GRMs with 5 to 35 K SNPs for the SNP groups with sequencing depth 0–10 (low), 20–30 (medium) and 40–60 (high). The smallest group of sequencing depth 40–60 had just over 35 K SNPs, so it was not possible to compare the different SNP groups at SNP numbers above 35 K. Table 3 present the narrow sense heritabilities from the genomic component $\left( {h_{G}^{2} } \right)$ for these different SNP sets, as well as the estimates obtained from using all SNPs in Table 2. Results show a tendency that use of smaller sets of SNPs has smaller effect in the high sequencing depth SNPs, e.g., using 15 K SNPs estimates in the high sequencing depth group are still close to those using all SNPs, while in the medium and lower sequencing-depth groups heritabilities are already lower using 35 K SNPs compared to using all SNPs.

**Table 3:** Estimated narrow sense heritabilities from genomic variance ($h_{G}^{2}$) for crown rust resistance and heading date, using genomic relationship matrices with different numbers of SNPs and sequence depths

# SNPs	Crown rust resistance			Heading date		
	Depth (0–10)	Depth (20–30)	Depth (40–60)	Depth (0–10)	Depth (20–30)	Depth (40–60)
5 K	0.04 (0.031)	0.12 (0.039)	0.17 (0.056)	0.02 (0.014)	0.03 (0.019)	0.08 (0.032)
10 K	0.04 (0.035)	0.12 (0.045)	0.21 (0.066)	0.03 (0.019)	0.08 (0.026)	0.11 (0.037)
15 K	0.07 (0.039)	0.13 (0.050)	0.31 (0.071)	0.01 (0.018)	0.08 (0.030)	0.19 (0.047)
20 K	0.11 (0.044)	0.19 (0.052)	0.26 (0.074)	0.02 (0.021)	0.10 (0.032)	0.18 (0.047)
25 K	0.11 (0.047)	0.19 (0.055)	0.30 (0.076)	0.03 (0.023)	0.08 (0.031)	0.21 (0.049)
30 K	0.11 (0.047)	0.21 (0.056)	0.34 (0.077)	0.05 (0.025)	0.08 (0.031)	0.21 (0.051)
35 K	0.13 (0.048)	0.18 (0.056)	0.33 (0.078)	0.06 (0.026)	0.08 (0.032)	0.22 (0.052)
All	0.18 (0.057)	0.25 (0.061)	0.33 (0.079)	0.07 (0.030)	0.11 (0.037)	0.22 (0.052)

Standard errors of heritabilities are in parentheses

### Simulation results

A simulation study was conducted to validate the estimation of genomic heritability when using GBS data with low depth. The results (Table 4) show that genomic heritability was under-estimated by more than half at depth 5 with estimated heritabilities around 0.10, and still shows some bias at high depth with estimates around 0.20, while the true value simulated was 0.25. When replicated phenotypes are available, the broad sense heritabilities were correctly estimated, but, as the narrow sense heritabilities were underestimated, this implies that the broad sense component incorrectly captures the part of additive variance that was not captured by the narrow sense heritability. With one replicate the environmental variance captures the part not explained by the genomic component at low depth (details not shown). Table 4 shows that there is no effect of the number of QTL affecting the trait in these simulated data sets.

**Table 4:** Estimated narrow sense heritability (*h* ^2^) in simulated data with one replicate per family (1rep), and narrow sense and broad sense heritability (*H* ^2^) with two replicates per family (2rep) with different numbers of QTL and different sequencing depths. Each scenario is based on 500 families and 1000 SNP markers and was repeated 50 times; standard error from the simulated replicates in parentheses. The true simulated narrow sense heritability was 0.25 and broad sense heritability 0.375

#QTL	Depth 5			Depth 15			Depth 25		
	1rep (*h* ^2^)	2rep (*h* ^2^)	2rep (*H* ^2^)	1rep (*h* ^2^)	2rep (*h* ^2^)	2rep (*H* ^2^)	1rep (*h* ^2^)	2rep (*h* ^2^)	2rep (*H* ^2^)
5	0.09 (0.008)	0.09 (0.006)	0.37 (0.006)	0.17 (0.011)	0.16 (0.008)	0.38 (0.005)	0.18 (0.012)	0.19 (0.009)	0.38 (0.006)
10	0.08 (0.008)	0.10 (0.006)	0.38 (0.005)	0.18 (0.009)	0.17 (0.008)	0.37 (0.006)	0.19 (0.011)	0.18 (0.009)	0.38 (0.006)
50	0.10 (0.006)	0.09 (0.004)	0.36 (0.005)	0.18 (0.010)	0.17 (0.007)	0.38 (0.005)	0.19 (0.011)	0.21 (0.008)	0.38 (0.005)
200	0.10 (0.007)	0.10 (0.006)	0.37 (0.004)	0.16 (0.010)	0.17 (0.008)	0.38 (0.005)	0.20 (0.013)	0.19 (0.008)	0.37 (0.005)
500	0.10 (0.009)	0.09 (0.005)	0.37 (0.006)	0.17 (0.012)	0.19 (0.006)	0.38 (0.005)	0.21 (0.011)	0.19 (0.007)	0.37 (0.005)
1000	0.10 (0.007)	0.10 (0.006)	0.37 (0.005)	0.18 (0.009)	0.17 (0.005)	0.38 (0.006)	0.21 (0.010)	0.19 (0.007)	0.37 (0.005)

## Discussion

In this study we present estimates of genomic heritabilities for two traits measured in pooled samples of families of perennial ryegrass based on pool-allele frequencies at variant positions obtained by genotyping-by-sequencing (GBS). This introduces a new approach that defines heritabilities not in individuals, but at the level of pool-samples and uses GBS to compute genomic relationships between pools. Very few studies have been published with heritabilities in ryegrass (Conaghan and Casler 2011; Elgersma 1990; Yamada et al. 2004), and largely these studies have quantified heritabilities based on pedigree information and phenotypes measured on individual (spaced) plants. For practical breeding, however, spaced individuals are of limited interest, because grasses are used in practice in densely sown swards. This is the first study that presents genomic heritabilities for traits measured on such swards, established as family-plots, and that uses GBS data to characterize relationships directly at the level of pooled families. To the best of our knowledge, the approach that we present here is novel. In order to validate the use of GBS and the computation of relationships between family-pools a large data set from a single year and single location was used; this implies that the heritabilities presented are ‘best case’ heritabilities that may include genotype-by-environment interactions.

Simulation studies indicated that genomic heritabilities are under-estimated at lower read depths (Table 4), and this was verified using empirical evidence (Table 2). Therefore, the most accurate estimates of narrow sense genomic heritability were with higher sequencing depth, and were 0.33 and 0.22 for crown rust resistance and heading date respectively. Variability in the size of heritability estimates was found when this study was compared to previous studies. Rust resistance is a quite variable trait in terms of classical heritability (Ravel and Charmet 1996); this diversity could be due to interaction with some other plant diseases or different experimental environments. In a recent study, Fè et al. (2015) presented family-based analysis of 1453 ryegrass F2 families produced in multiple years and locations in Europe, and promising broad sense heritabilities were determined for various traits measured. In the case of rust resistance, they found 0.26 and 0.34 broad sense heritabilities within and across parental populations, which are lower than those found here. Generally, heading date is a highly heritable trait in several plant species. The estimates of genomic heritability for heading date presented in Fè et al. (2015) were very similar to our results.

Results summarized in Table 1 and Fig. 1, revealed that diagonals of GRMs were inflated when using SNPs with low depth. In contrast off-diagonals are little affected by sequencing depth. The higher diagonal values can be interpreted as (falsely) showing a higher inbreeding level in the samples, which is consistent with low-depth sequencing falsely obtaining homozygote genotypes, which are in reality heterozygote. However, pool samples have a larger range of true pool-frequencies, e.g., in biparental pools in quarters, and also miss-genotyping between heterozygotes contributes to the inflated GRM diagonals, as is shown by biases still being present at sequencing depth 25. Simulation results (Table 4) revealed that estimates of genomic heritability are biased downwards at lower sequencing depth. It has already been shown that, when using GBS data, estimates of allele effects in a Genome Wide Association Study (GWAS) are subject to bias because of low sequencing depth (Ashraf et al. 2014). This bias leads to an underestimation of the allele substitution effect. Measurement error on covariates is well known to cause underestimation of regression coefficients (Chesher 1991) in least squares or fixed effect models. Our simulation results indicate that also variance components in random effect models are under-estimated, and the amount of under-estimation corresponds to the square of the bias terms on allele effects given by Ashraf et al. (2014). This is consistent because genomic variances relate to the square of allele effects. Ashraf et al. (2014) also considered the effect of sequencing errors in the framework of GWAS, and showed that sequencing error further biased downwards allele effect estimates. When estimating genomic heritabilities the same effect would be expected that sequencing error gives an additional downward bias in genomic heritability estimates.

To determine if the effects of sequencing depth that we observe (Table 2) are influenced by different SNP numbers in each group, we sampled equal numbers of SNPs within each sequencing depth (Table 3). This shows tendencies that reducing SNP number within low sequencing depth SNPs has stronger effect, which adds an additional downward bias when using low sequencing depth. However, the effects of reducing SNP numbers could only be studied up to 35 K SNPs, which was the size of the smallest SNP group. In the main analyses, the low sequencing depth groups had all well over 100 K SNPs, and therefore we believe that our main results are not much influenced by differences in SNP number between the SNP groups. In other contexts it has also been shown that variance explained by SNPs depends on the number of SNPs used (e.g., Yang et al. 2010, Jensen et al. 2012). In the context of using GBS these results suggest that the combination of low sequencing depth and low number of SNPs may need to be avoided.

The traits studied here, crown rust resistance and heading date, are commonly assumed to have relatively simpler genetic architectures. Our simulation results did not show differences between genetic architectures based on 5 to 1000 QTL, which suggests that our results can be generalized to also apply to more polygenic traits.

## Electronic supplementary material

Supplementary material 1 (DOCX 174 kb)
