# Supplementary material for: Estimating genomic heritabilities at the level of family-pool samples of perennial ryegrass using genotyping-by-sequencing
Source: Theor Appl Genet. 2015 Sep 25;129:45–52. doi: 10.1007/s00122-015-2607-9 (PMC4703624; doi:10.1007/s00122-015-2607-9)
Supplement: Supplementary file 1 — Supplementary material 1 (DOCX 174 kb) [file 122_2015_2607_MOESM1_ESM.docx]

**Supplementary material**

**Estimating genomic heritabilities at the level of family-pool samples of perennial ryegrass using genotyping-by-sequencing**

Bilal Ashraf^1^, Stephen Byrne^2,^ Dario Fé^13^, Adrian Czaban^2^, Torben Asp^2^, Morten G. Pedersen^3^, Ingo Lenk^3^, Niels Roulund^3^, Thomas Didion^3^, Christian S. Jensen^3^, Just Jensen^1^, Luc L. Janss^1^*

1-Dept. of Molecular Biology and Genetics, Aarhus University, Blichers Allé 20, postbox 50, 8830

Tjele, Denmark. 2-Dept. of Molecular Biology and Genetics, Aarhus University, Forsøgsvej 1, 4200

Slagelse, Denmark. 3- DLF-Trifolium A/S, Research Division, Højerupvej 31, 4600 Store Heddinge, Denmark.

* Author to whom correspondence should be addresses; E-mail: (luc.janss@mbg.au.dk); Tel.: +45 87158008; Fax: +45 87154994.

Journal name: Theoretical and Applied Genetics (TAG)


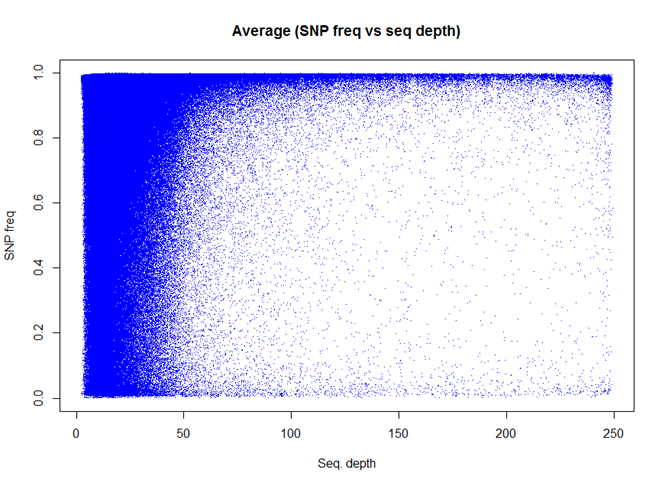


Fig S1: Average SNP frequency against average SNP sequencing depth of unedited SNP markers. Final editing removed SNPs with allele frequencies less than 0.02 and greater than 0.98, and removed SNPs with depth above 60. This figure show the original SNP data before splitting into sub groups, the numbers of SNPs in each group are listed in Table 1.

**Supplemental Note 1**

**Initial data processing**

Adaptors were removed from the 3’ end of reads using fastx_clipper (<http://hannonlab.cshl.edu/fastx_toolkit/index.html>, V 0.0.13), and reads with less than 40 bp remaining after adaptor removal were discarded. Poor quality bases were trimmed from the 3’ end of reads using sickle (Joshi and Fass, 2011, V 1.000). This slides a window (10% of read length) across the read and trims bases when the average quality of a window falls below 20. Family samples were demultiplexed using sabre (<https://github.com/najoshi/sabre>, V 1.000) and no mismatches were allowed within the barcode sequence. Identical families from multiple sequencing runs were concatenated. The distribution of read numbers across the 995 F2 families is shown in Figure SN1.1.

***
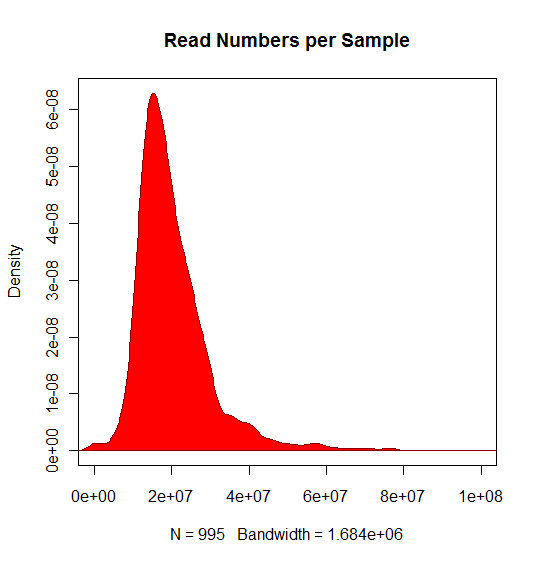
***

Figure SN1.1: Density plot of read numbers per F2 family after initial data processing.

**Draft reference assembly**

We used a draft sequence assembly of the perennial ryegrass genome in order to anchor the reads. This consisted of 42,426 scaffolds greater than 10 Kb with a total scaffold length of 904 Mb. The average scaffold length was 21,315 bp.

**SNP calling pipeline**

Reads were aligned to the draft assembly using BWA ([Li and Durbin 2009](#_ENREF_1)) V 0.7.8-r455, and the resulting SAM files were converted to BAM files and sorted by coordinate using Picard Tools (<https://github.com/broadinstitute/picard>, V 1.87). A module within GATK ([McKenna et al. 2010](#_ENREF_2)) (release 47760) was used to identify a list of potential indel sites (RealignerTargetCreator), and local re-alignment was performed around these sites (IndelRealigner). This was performed on a per sample basis. Putative SNPs were identified across the 995 F2 families using UnifiedGenotyper with sample ploidy set to four. The VCF file was first filtered for sites where the root mean square mapping quality was at least 30, and the minor allele frequency was at least 0.01. Minor allele frequency estimates included genotype calls based on one or more reads. There were 1,020,065 SNPs meeting these criteria.

**Allele frequencies in each F2 family**

The frequency of the variant allele was calculated for each F2 family at all 1,020,065 SNP positions (variant frequency = variant allele count/(reference allele count+variant allele count)). Allele frequencies were calculated when there was one or more reads mapping. Final editing steps were based on sample missing rate, SNP frequency and SNP depth as described in the main manuscript, and reduced the data to 990 families and 728,359 SNPs.

**References**

Joshi NA, Fass JN. (2011). Sickle: A sliding-window, adaptive, quality-based trimming tool for FastQ files (Version 1.33) [Software]. Available at https://github.com/najoshi/sickle.

Li H, Durbin R (2009) Fast and accurate short read alignment with Burrows–Wheeler transform. Bioinformatics 25:1754-1760

McKenna A, Hanna M, Banks E, Sivachenko A, Cibulskis K, Kernytsky A, Garimella K, Altshuler D, Gabriel S, Daly M (2010) The Genome Analysis Toolkit: a MapReduce framework for analyzing next-generation DNA sequencing data. Genome research 20:1297-1303
